# Emerging racial disparities among Medicare beneficiaries and Veterans with metastatic castration-sensitive prostate cancer

**DOI:** 10.1038/s41391-024-00815-1

**Published:** 2024-04-02

**Authors:** Daniel J. George, Neeraj Agarwal, Krishnan Ramaswamy, Zachary Klaassen, Rhonda L. Bitting, David Russell, Rickard Sandin, Birol Emir, Hongbo Yang, Wei Song, Yilu Lin, Agnes Hong, Wei Gao, Stephen J. Freedland

**Affiliations:** 1 Department of Medicine, Duke Cancer Institute, Duke University School of Medicine, Durham, NC USA; 2 Department of Internal Medicine, Huntsman Cancer Institute, University of Utah, Salt Lake City, UT USA; 3 Pfizer Inc., New York, NY USA; 4 Department of Surgery, Medical College of Georgia at Augusta University, Georgia Cancer Center, Augusta, GA USA; 5 Department of Medicine, Durham VA Medical Center, Durham, NC USA; 6 Pfizer AB, Sollentuna, Sweden; 7 Analysis Group, Inc., Boston, MA USA; 8 Department of Health Policy and Management, Tulane University, New Orleans, LA USA; 9 Formerly of Astellas Pharma Inc., Northbrook, IL USA; 10 Section of Urology, Durham VA Medical Center, Durham, NC USA; 11 Department of Urology, Samuel Oschin Comprehensive Cancer Institute, Cedars-Sinai Medical Center, Los Angeles, CA USA

**Keywords:** Cancer therapy, Prostate cancer, Cancer epidemiology

## Abstract

**Background:**

Previous studies have shown that Black men receive worse prostate cancer care than White men. This has not been explored in metastatic castration-sensitive prostate cancer (mCSPC) in the current treatment era.

**Methods:**

We evaluated treatment intensification (TI) and overall survival (OS) in Medicare (2015–2018) and Veterans Health Administration (VHA; 2015–2019) patients with mCSPC, classifying first-line mCSPC treatment as androgen deprivation therapy (ADT) + novel hormonal therapy; ADT + docetaxel; ADT + first-generation nonsteroidal antiandrogen; or ADT alone.

**Results:**

We analyzed 2226 Black and 16,071 White Medicare, and 1020 Black and 2364 White VHA patients. TI was significantly lower for Black vs White Medicare patients overall (adjusted odds ratio [OR] 0.68; 95% confidence interval [CI] 0.58–0.81) and without Medicaid (adjusted OR 0.70; 95% CI 0.57–0.87). Medicaid patients had less TI irrespective of race. OS was worse for Black vs White Medicare patients overall (adjusted hazard ratio [HR] 1.20; 95% CI 1.09–1.31) and without Medicaid (adjusted HR 1.13; 95% CI 1.01–1.27). OS was worse in Medicaid vs without Medicaid, with no significant OS difference between races. TI was significantly lower for Black vs White VHA patients (adjusted OR 0.75; 95% CI 0.61–0.92), with no significant OS difference between races.

**Conclusions:**

Guideline-recommended TI was low for all patients with mCSPC, with less TI in Black patients in both Medicare and the VHA. Black race was associated with worse OS in Medicare but not the VHA. Medicaid patients had less TI and worse OS than those without Medicaid, suggesting poverty and race are associated with care and outcomes.

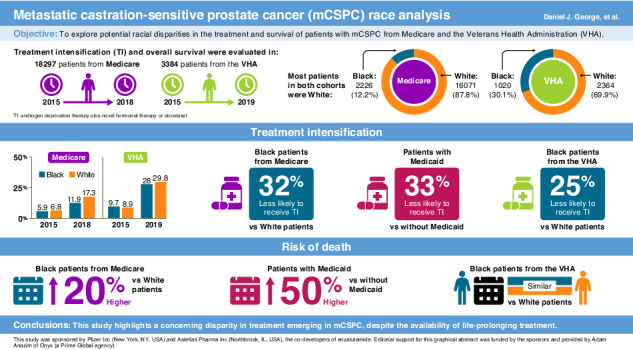

## Introduction

The treatment landscape for metastatic castration-sensitive prostate cancer (mCSPC) has rapidly evolved. Treatment intensification (TI) with docetaxel, novel hormonal therapy (NHT; abiraterone, apalutamide, enzalutamide), or both, added to androgen deprivation therapy (ADT) has substantially improved survival [1–9] and is a consensus guideline recommendation [10–13]. However, TI is underutilized in favor of ADT alone or with first-generation nonsteroidal anti-androgen (NSAA) [14–21], despite guidelines recommending first-generation NSAAs only to block testosterone flare [11, 13]. Reasons are not well understood but may include disease characteristics or comorbidities, cost or access issues, practice pattern inertia, ignorance of current data, or safety and tolerability perceptions [22].

Previous studies found that Black men are more likely to receive inadequate prostate cancer (PC) care than White men [23–30]; however, this has not been explored during the NHT era for mCSPC. This is particularly concerning because Black men have higher PC mortality and are more likely to develop aggressive disease at a younger age [31–37]. While the latter may be due to biologic or genetic factors [38, 39], the former is driven in part by factors affecting access to care [40–43], partly resulting from systemic racism. In clinical trials, there are often too few Black patients to analyze outcomes by race or race is not reported at all [44]. As we progress further into the NHT era, we hypothesize that the disparities evident in the treatment and survival of Black men, compared with White men with mCSPC, remain.

Real-world data are vital to understanding racial disparities in mCSPC. We evaluated potential disparities in the treatment and survival of men with mCSPC in the USA. We used two large, nationally representative USA claims databases with different treatment settings and payer structures: Medicare, which includes supplemental plan options and dual enrollment with Medicaid for low-income patients, and the Veterans Health Administration (VHA), a single-payer, equal-access, closed system. This is the first large study of racial disparities in treatment, survival, and associated factors, including access to care and poverty, in mCSPC during the NHT era.

## Methods

### Data sources

Data were collected from a 100% sample of Medicare Fee-For-Service beneficiaries (January 1, 2015 to December 31, 2018) from the Centers for Medicare and Medicaid Services and the VHA Corporate Data Warehouse (January 1, 2015 to June 22, 2019). Medicare data contain patient demographics, enrollment, and claims history (including drug, diagnosis, physician visits, procedures [Medicare Part A, B, D]). Death dates were verified against US Social Security Administration agency or Railroad Retirement Board records. VHA data comprise electronic records from the largest integrated healthcare system in the USA with approximately 1300 care sites serving >9 million veterans and their dependents annually [45], and contain patient demographics, enrollment, and clinical information (e.g., inpatient and outpatient pharmacy data, laboratory tests, hospitalizations, outpatient visits). Death dates were verified through VHA facilities, death certificates, and the National Cemetery Administration. Institutional Review Board (IRB) exemption was obtained from the New England IRB (Medicare) and from Southeast Louisiana Veterans Health Care System IRB (VHA).

### Study cohort

Unless specified, the same sample selection criteria were applied to Medicare and VHA patients with metastatic PC to select mCSPC patients (Supplementary Fig. S1). Patients had ≥1 medical claim with a diagnosis code for PC and ≥1 claim for metastasis on or after the first observed PC diagnosis. Patients received ADT (surgical or medical castration) <90 days prior to or any time after the first observed metastasis diagnosis.

The first ADT initiation date meeting these requirements was defined as the index date. Adult Black, non-Hispanic, and White males at index with continuous enrollment ≥365 days before (baseline period) and ≥120 days after index were included. Follow-up was from index to end of continuous enrollment, data availability, or death, whichever occurred first.

Exclusion criteria are shown in Supplementary Fig. S1; because treatment options for mCSPC have changed substantially since 2014 (with the introduction of docetaxel and NHT), we excluded patients with index dates before 2015. Patients with missing race information and VHA patients with missing prostate-specific antigen (PSA) measurements <120 days before index were excluded. PSA information is unavailable in Medicare. Included patients were classified into Black and White groups based on information reported during index year (Supplementary Fig. S1).

### Patient characteristics and study outcomes

Demographics, baseline disease characteristics, treatment history, and baseline comorbidities (modified Charlson Comorbidity Index [CCI; excluding cancer] and relevant individual comorbidities) [46, 47] were assessed. First-line mCSPC treatment was defined as treatment received <30 days before and <120 days after index (index window) and classified into ADT + NHT, ADT + docetaxel, ADT + first-generation NSAA, and ADT alone. NSAA use was required for ≥90 days to avoid capturing short-term use for testosterone flare. Patients who received NHT or docetaxel and NSAA were categorized into ADT + NHT or ADT + docetaxel groups. Patients who received both NHT and docetaxel during the index window were categorized based on the drug received first.

TI was defined as receiving ADT + docetaxel or ADT + NHT as first-line mCSPC treatment. Overall survival (OS) was defined as time from index to death. Patients alive at the end of continuous eligibility or data availability were censored.

### Statistical analyses

Statistical analyses were conducted by race in Medicare and VHA data separately. Summary statistics were reported for continuous variables. Counts and percentages were reported for categorical variables. Standardized mean differences (SMDs) were calculated for unadjusted comparisons of baseline characteristics. SMDs >10% were considered statistically significant [48–50].

The proportion of Black and White patients who received ADT + NHT, ADT + docetaxel, ADT + NSAA, and ADT alone annually was determined. Unadjusted and adjusted generalized linear models (GLMs) with binomial distribution and logit link estimated the odds ratio (OR) of TI. Adjusted models accounted for baseline demographics, disease characteristics, treatment history, and comorbidities and were selected based on clinical input and SMD > 10%. In the Medicare analysis, a second adjusted model accounted for dual Medicaid enrollment. Medicaid enrollment was recorded based on income and assets; from 2015 to 2020, the median income eligibility limit was 138% of the Federal Poverty Level [51]. For the VHA, a second adjusted model accounted for median household income per zip code (patient-level data were unavailable). For sensitivity analyses, VHA models accounted for baseline laboratory values (i.e., PSA, hemoglobin, alkaline phosphatase) and education attainment (i.e., percentage of population in a zip code age ≥25, with a bachelor’s degree or higher).

Kaplan–Meier analyses assessed OS. Unadjusted and adjusted Cox proportional hazards (PH) models estimated the hazard ratio (HR). Adjusted Cox PH models accounted for the same variables used in the GLMs for TI.

## Results

### Patient population

Overall, 2226 (12.2%) Black and 16,071 (87.8%) White patients were included in the Medicare analysis; 12.8% were also Medicaid-enrolled; 40.3% of Black and 9.0% of White patients in Medicare were Medicaid-enrolled. The VHA analysis included 1020 (30.1%) Black and 2364 (69.9%) White patients (Table 1). Black patients were younger than White patients (mean age, Medicare: 73.9 vs 76.9 years, SMD –38.3%; VHA: 70.1 vs 74.4 years, SMD –45.2%). A higher proportion of Black vs White patients resided in the South (Medicare: 55.0% vs 33.5%, SMD 44.4%; VHA: 38.5% vs 29.4%, SMD 19.3%). Proportions of Black and White patients with visceral metastasis or on pain medication at baseline were similar. In the VHA, more Black vs White patients had visceral metastasis (12.5% vs 8.7%, SMD 12.6%) and pain medication use (75.1% vs 62.2%; SMD 28.0%). Black patients had a higher mean modified CCI score vs White patients in both datasets. In the VHA, Black patients had a higher median PSA (40.5 vs 23.6; Supplementary Table S1), lower median hemoglobin (12.4 vs 13.3), and similar median alkaline phosphatase measurements vs White patients.

**Table 1:** Patient baseline demographics and clinical characteristics.

	Medicare				VHA			
	Overall (*N* = 18,297)	Black patients (*n* = 2226)	White patients (*n* = 16,071)	Black vs White patients SMD, %	Overall (*N* = 3384)	Black patients (*n* = 1020)	White patients (*n* = 2364)	Black vs White patients SMD, %
Age at index date, mean ± SD (median) [IQR], years	76.6 ± 7.8 (75.8) [70.8–82.0]	73.9 ± 8.5 (73.7) [68.4–79.4]	76.9 ± 7.6 (76.1) [71.2–82.4]	–38.31	73.1 ± 9.4 (71.7) [67.0–80.0]	70.1 ± 9.7 (69.0) [63.4–75.9]	74.4 ± 9.0 (72.7) [68.4–81.3]	–45.22
Age group at index date, *n* (%), years								
≤59	297 (1.6)	122 (5.5)	175 (1.1)	24.83	246 (7.3)	131 (12.8)	115 (4.9)	28.37
60–69	3533 (19.3)	616 (27.7)	2917 (18.2)	22.80	1136 (33.6)	436 (42.7)	700 (29.6)	27.59
70–79	8540 (46.7)	977 (43.9)	7563 (47.1)	–6.37	1158 (34.2)	274 (26.9)	884 (37.4)	–22.70
≥80	5927 (32.4)	511 (23.0)	5416 (33.7)	–24.02	844 (24.9)	179 (17.5)	665 (28.1)	–25.41
Index year, *n* (%)								
2015	4065 (22.2)	537 (24.1)	3528 (22.0)	5.16	525 (15.5)	154 (15.1)	371 (15.7)	–1.65
2016	4893 (26.7)	618 (27.8)	4275 (26.6)	2.61	536 (15.8)	171 (16.8)	365 (15.4)	3.60
2017	5357 (29.3)	624 (28.0)	4733 (29.5)	–3.13	690 (20.4)	211 (20.7)	479 (20.3)	1.05
2018	3982 (21.8)	447 (20.1)	3535 (22.0)	–4.70	780 (23.0)	234 (22.9)	546 (23.1)	–0.37
2019	NA	NA	NA	NA	853 (25.2)	250 (24.5)	603 (25.5)	–2.30
Geographic region^a^, *n* (%)								
South	6605 (36.1)	1224 (55.0)	5381 (33.5)	44.35	1089 (32.2)	393 (38.5)	696 (29.4)	19.28
Midwest	4549 (24.9)	464 (20.8)	4085 (25.4)	–10.86	900 (26.6)	284 (27.8)	616 (26.1)	4.03
West	3554 (19.4)	169 (7.6)	3385 (21.1)	–39.18	896 (26.5)	190 (18.6)	706 (29.9)	–26.45
Northeast	3589 (19.6)	369 (16.6)	3220 (20.0)	–8.95	499 (14.7)	153 (15.0)	346 (14.6)	1.02
Medicaid enrollment, *n* (%)	2339 (12.8)	898 (40.3)	1441 (9.0)	78.16	NA	NA	NA	NA
Mean income^b^, mean ± SD (median) [IQR], USD	70,457 ± 30,178 (62,824) [49,675–84,787]	54,383 ± 24,646 (48,406) [37,045–65,631]	72,665 ± 30,200 (64,311) [51,449–86,648]	–66.3	61,893 ± 23,523 (57,132) [45,969–73,075]	54,230 ± 21,392 (50,260) [39,412–64,626]	65,173 ± 23,635 (60,134) [49,347–76,294]	–48.6
Percent of population age ≥25 years with bachelor’s degree^b^, mean ± SD (median) [IQR]	33.8 ± 18.1 (29.8) [19.4–45.4]	26.7 ± 15.7 (22.5) [15.3–34.0]	34.8 ± 18.2 (31.1) [20.0–46.7]	–47.8	28.5 ± 14.8 (25.0) [17.5–36.1]	26.6 ± 14.3 (23.5) [16.1–33.6]	29.3 ± 14.9 (25.7) [18.3–37.6]	–19.0
Site of metastasis^c^, *n* (%)								
Bone only	9596 (52.4)	1186 (53.3)	8410 (52.3)	1.90	1574 (46.5)	457 (44.8)	1117 (47.3)	–4.91
Bone and node only	1207 (6.6)	177 (8.0)	1030 (6.4)	5.98	155 (4.6)	47 (4.6)	108 (4.6)	0.19
Node only	2860 (15.6)	298 (13.4)	2562 (15.9)	–7.23	211 (6.2)	74 (7.3)	137 (5.8)	5.91
Viscera	1925 (10.5)	249 (11.2)	1676 (10.4)	2.44	333 (9.8)	128 (12.5)	205 (8.7)	12.61
Other	2709 (14.8)	316 (14.3)	2393 (14.9)	6.27	1111 (32.8)	314 (30.8)	797 (33.7)	–6.27
Time from the first observed metastasis to index date, mean ± SD (median) [IQR], days	245.8 ± 549.7 (21.0) [7.0–105.0]	225.1 ± 517.1 (26.0) [8.0–104.0]	248.9 ± 554.3 (21.0) [7.0–106.0]	–4.44	188.5 ± 538.5 (16.0) [3.0–57.0]	203.1 ± 595.1 (17.5) [3.5–54.0]	182.1 ± 511.8 (16.0) [2.0–58.0]	3.79
Previously untreated prostate cancer^d^, *n* (%)	11,924 (65.2)	1490 (66.9)	10,434 (64.9)	4.25	2367 (69.9)	692 (67.8)	1675 (70.9)	–6.54
Treatment history, *n* (%)								
Pain medication	9575 (52.3)	1244 (55.9)	8331 (51.8)	8.12	2237 (66.1)	766 (75.1)	1471 (62.2)	28.02
Corticosteroids	396 (2.2)	31 (1.4)	365 (2.3)	–6.55	69 (2.0)	19 (1.9)	50 (2.1)	–1.81
Bone protective agents	376 (2.1)	32 (1.4)	344 (2.1)	–5.31	52 (1.5)	13 (1.3)	39 (1.6)	–3.13
Radiotherapy	599 (3.3)	55 (2.5)	544 (3.4)	–5.42	89 (2.6)	41 (4.0)	48 (2.0)	11.63
Prostatectomy	1660 (9.1)	179 (8.0)	1481 (9.2)	–4.18	236 (7.0)	80 (7.8)	156 (6.6)	4.81
Modified CCI (excluding cancer), mean ± SD (median) [IQR]	2.6 ± 2.5 (1.7) [0.0–4.0]	3.4 ± 2.8 (2.9) [1.3–5.0]	2.5 ± 2.4 (1.7) [0.0–3.7]	34.39	1.8 ± 1.9 (1.3) [0.0–2.9]	1.9 ± 2.1 (1.3) [0.0–2.9]	1.7 ± 1.8 (1.3) [0.0–2.9]	10.51
Comorbidities, *n* (%)								
Hypertension	14,581 (79.7)	1950 (87.6)	12,631 (78.6)	24.21	2451 (72.4)	789 (77.4)	1662 (70.3)	16.09
Hyperlipidemia	12,881 (70.4)	1406 (63.2)	11,475 (71.4)	–17.63	1803 (53.3)	466 (45.7)	1337 (56.6)	–21.88
Diabetes	5839 (31.9)	1024 (46.0)	4815 (30.0)	33.51	1120 (33.1)	362 (35.5)	758 (32.1)	7.25
Chronic obstructive pulmonary disease	3468 (19.0)	506 (22.7)	2962 (18.4)	10.65	549 (16.2)	126 (12.4)	423 (17.9)	–15.51
Arrhythmia	2919 (16.0)	354 (15.9)	2565 (16.0)	–0.16	171 (5.1)	46 (4.5)	125 (5.3)	–3.60
Congestive heart failure	2878 (15.7)	456 (20.5)	2422 (15.1)	14.20	333 (9.8)	100 (9.8)	233 (9.9)	–0.18
Stroke	2312 (12.6)	282 (12.7)	2030 (12.6)	0.11	170 (5.0)	44 (4.3)	126 (5.3)	–4.75
Obesity	3016 (16.5)	376 (16.9)	2640 (16.4)	1.25	501 (14.8)	149 (14.6)	352 (14.9)	–0.80
Myocardial infarction	1735 (9.5)	219 (9.8)	1516 (9.4)	1.37	95 (2.8)	24 (2.4)	71 (3.0)	–4.03
Acute coronary syndrome	722 (3.9)	114 (5.1)	608 (3.8)	6.49	47 (1.4)	12 (1.2)	35 (1.5)	−2.66
Angina pectoris	673 (3.7)	87 (3.9)	586 (3.6)	1.37	63 (1.9)	20 (2.0)	43 (1.8)	1.04

*ADT* androgen deprivation therapy, *CCI* Charlson Comorbidity Index, *IQR* interquartile range, *NA* not applicable, *PSA* prostate-specific antigen, *SD* standard deviation, *SMD* standardized mean difference, *USD* US dollars, *VHA* Veterans Health Administration.

^a^The geographic region documented during the index year was used. Midwest includes IL, IN, IA, KS, MI, MN, MO, NE, ND, OH, SD, and WI; Northeast includes CT, ME, MA, NH, NJ, NY, PA, RI, and VT; South includes AL, AR, DE, DC, FL, GA, KY, LA, MD, MS, NC, OK, SC, TN, TX, VA, and WV; West includes AK, AZ, CA, CO, HI, ID, MT, NV, NM, OR, UT, WA, and WY.

^b^Zip-code level information from the American Community Survey 2019 5-year estimates for median income and the percentage of people in a zip code aged 25+ years with at least a bachelor’s degree was used.

^c^All metastases prior to the index date and within 90 days after the index date were included. Patients with visceral metastasis may or may not have bone and/or node metastasis. Other sites of metastasis included other specified sites such as urinary organs, genital organs, and kidney, as well as unspecified sites.

^d^Previously untreated prostate cancer was defined as no evidence of ADT treatment, radiation therapy, or surgery for prostate cancer any time before the index date.

### Treatment intensification

**Fig. 1:**
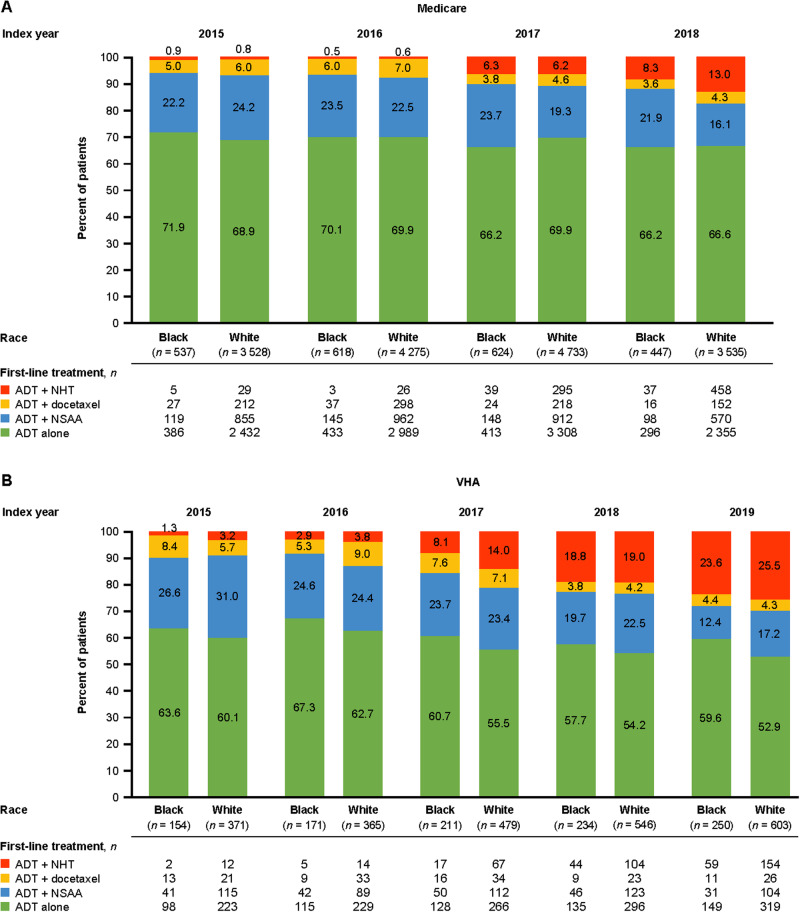
First-line treatment for mCSPC over time by race. **A** First-line treatment for mCSPC over time by race in Medicare. **B** First-line treatment for mCSPC over time by race in the VHA. ADT androgen deprivation therapy, mCSPC metastatic castration-sensitive prostate cancer, NHT novel hormonal therapy, NSAA nonsteroidal antiandrogen, VHA Veterans Health Administration.

The proportion of patients receiving TI as first-line mCSPC treatment increased slowly over time (Fig. 1): Medicare, from 5.9% in 2015 to 11.9% in 2018 for Black patients and from 6.8% to 17.3% for White patients; VHA, from 9.7% in 2015 to 28.0% in 2019 for Black patients and from 8.9% to 29.8% for White patients. However, TI was overall underutilized (Medicare: 10.3%; VHA: 19.9%). Even in 2018 and 2019, the most recent years of data available, more than two-thirds of patients did not receive upfront NHT or docetaxel, across both races.

For Medicare, the unadjusted odds of Black patients receiving TI was 21% lower vs White patients (Table 2; unadjusted OR 0.79, 95% confidence interval [CI] 0.67–0.92, *p* = 0.003; adjusted OR 0.68, 95% CI 0.58–0.81, *p* < 0.001). Among patients without Medicaid enrollment, adjusted odds were 30% lower for Black vs White patients (adjusted OR 0.70, 95% CI 0.57–0.87, *p* = 0.001). Among patients with Medicaid enrollment, there was no significant difference in TI between races. Overall, Medicaid-enrolled patients were less likely to receive TI compared with those not Medicaid-enrolled (Table 3; adjusted OR 0.67, 95% CI 0.57–0.80, *p* < 0.001; Supplementary Fig. S2).

**Table 2:** Rate of first-line TI and Cox proportional hazards models for OS among Black vs White patients with mCSPC.

Medicare			VHA		
**First-line TI**^**a**^ **in Black vs White patients, OR (95% CI)**^**b**^		***P*-value**	**First-line TI**^**a**^ **in Black vs White patients, OR (95% CI)**		***P*-value**
Unadjusted	0.79 (0.67–0.92)	0.003*	Unadjusted	0.86 (0.71–1.03)	0.094
Adjusted model 1^c^	0.68 (0.58–0.81)	< 0.001*	Adjusted model 2^c^	0.75 (0.61–0.92)	0.006*
Adjusted model 1^c^ + adjusting for Medicaid enrollment (among all patients)	0.78 (0.64–0.90)	0.002*	Adjusted model 2^c^ + adjusting for median household income per zip code	0.77 (0.63–0.95)	0.016*
Adjusted model 1^c^ + adjusting for Medicaid enrollment and its interaction with race					
Without Medicaid enrollment	0.70 (0.57–0.87)	0.001*			
With Medicaid enrollment	0.89 (0.65–1.20)	0.438			
**OS in Black vs White patients, HR (95% CI)**^**d**^		***P*-value**	**OS in Black vs White patients, HR (95% CI)**		***P*-value**
Unadjusted	1.26 (1.16–1.37)	< 0.001*	Unadjusted	1.0 (0.89–1.13)	0.989
Adjusted model 1^c^	1.20 (1.09–1.31)	< 0.001*	Adjusted model 2^c^	1.07 (0.94–1.22)	0.295
Adjusted model 1^c^ + adjusting for Medicaid enrollment (among all patients)	1.06 (0.96–1.16)	0.241	Adjusted model 2^c^ + adjusting for median household income per zip code	1.07 (0.94–1.22)	0.315
Adjusted model 1^c^ + adjusting for Medicaid enrollment and its interaction with race					
Without Medicaid enrollment	1.13 (1.01–1.27)	0.037*			
With Medicaid enrollment	0.95 (0.82–1.10)	0.479			

*CI* confidence interval, *HR* hazard ratio, *mCSPC* metastatic castration-sensitive prostate cancer, *OR* odds ratio, *OS* overall survival, *TI* treatment intensification, *VHA* Veterans Health Administration.

^a^Intensification of first-line treatment for mCSPC was defined as treatment with androgen deprivation therapy + novel hormonal therapy or androgen deprivation therapy + docetaxel.

^b^Interaction between race and Medicaid enrollment: *p* = 0.220.

^c^The adjusted models 1 and 2 accounted for index age, index year, site of metastasis, geographic region, race, time from metastasis to index date, previously untreated prostate cancer, use of pain medication, modified Charlson Comorbidity Index (excluding cancer), previous diagnosis of hypertension, hyperlipidemia, diabetes, chronic obstructive pulmonary disease, and congestive heart failure, and a composite variable of previous diagnosis with myocardial infarction, acute coronary syndrome, or angina pectoris.

^d^Interaction between race and Medicaid enrollment: *p* = 0.064.

**Table 3:** Rate of first-line TI and Cox proportional hazards models for OS among Medicare patients with mCSPC with vs without Medicaid enrollment.

Medicare		
**First-line TI**^**a**^ **in patients with vs without Medicaid enrollment, OR (95% CI)**^**b**^		*P*-value
Adjusted model 1^c^ + adjusting for Medicaid enrollment overall (among all patients)	0.67 (0.57–0.80)	0.001*
Adjusted model 1^c^ + adjusting for Medicaid enrollment and its interaction with race		
Among Black patients	0.80 (0.58–1.10)	0.165
Among White patients	0.63 (0.52–0.77)	< 0.001*
**OS in patients with vs without Medicaid enrollment, HR (95% CI)**^d^		
Adjusted model 1^c^ + adjusting for Medicaid enrollment (among all patients)	1.50 (1.37–1.63)	< 0.001*
Adjusted model 1^c^ + adjusting for Medicaid enrollment and its interaction with race		
Among Black patients	1.49 (1.31–1.69)	< 0.001*
Among White patients	1.57 (1.42–1.74)	< 0.001*

*CI* confidence interval, *HR* hazard ratio, *mCSPC* metastatic castration-sensitive prostate cancer, *OR* odds ratio, *OS* overall survival, *TI* treatment intensification.

^a^Intensification of first-line treatment for mCSPC was defined as treatment with androgen deprivation therapy + novel hormonal therapy or androgen deprivation therapy + docetaxel.

^b^Interaction between race and Medicaid enrollment: *p* = 0.220.

^c^The adjusted models 1 and 2 accounted for index age, index year, site of metastasis, geographic region, race, time from metastasis to index date, previously untreated prostate cancer, use of pain medication, modified Charlson Comorbidity Index (excluding cancer), previous diagnosis of hypertension, hyperlipidemia, diabetes, chronic obstructive pulmonary disease, and congestive heart failure, and a composite variable of previous diagnosis with myocardial infarction, acute coronary syndrome, or angina pectoris.

^d^Interaction between race and Medicaid enrollment: *p* = 0.064.

For the VHA, the unadjusted odds of Black patients receiving TI was not statistically significant (Table 2). After adjusting for patient characteristics, a statistically significant 25% lower TI rate among Black patients was observed (adjusted OR 0.75, 95% CI 0.61–0.92, *p* = 0.006; additionally accounting for median household income per zip code also showed a difference: OR 0.77, 95% CI 0.63–0.95, *p* = 0.016). Sensitivity analyses showed similar results (Supplementary Table S2). The distribution of subsequent treatment was similar between Black and White patients in both datasets (Supplementary Table S3).

### Overall survival

**Fig. 2:**
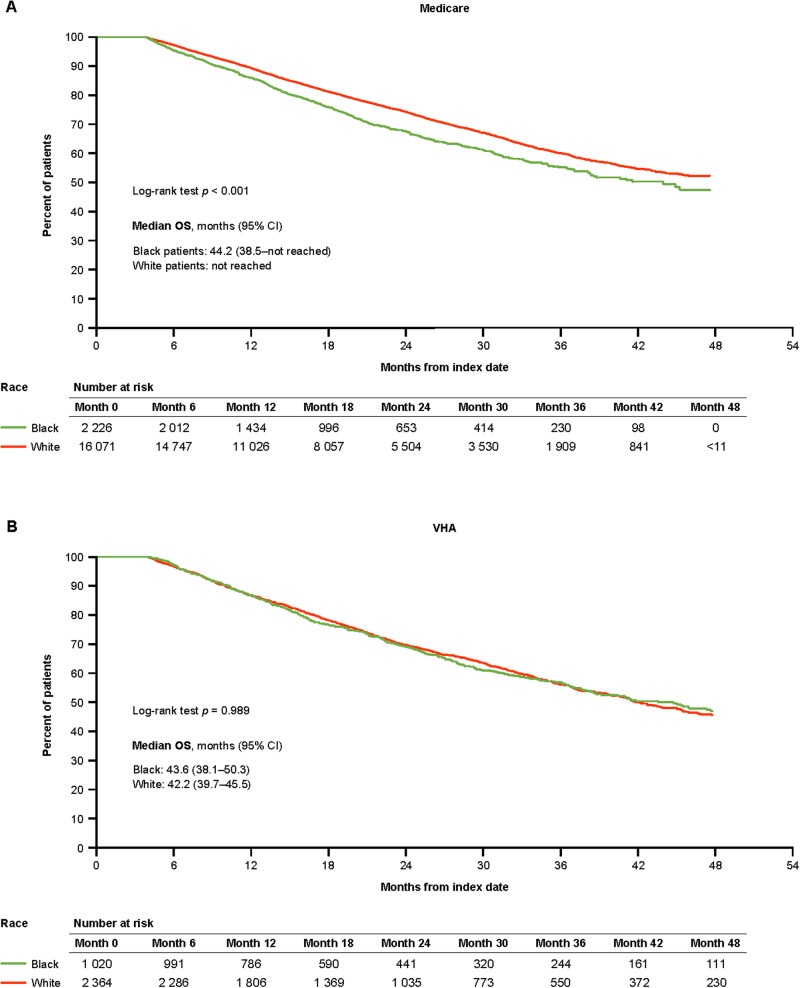
OS among patients with mCSPC by race. **A** OS among patients with mCSPC by race in Medicare. **B** OS among patients with mCSPC by race in the VHA. CI confidence interval, mCSPC metastatic castration-sensitive prostate cancer, OS overall survival, VHA Veterans Health Administration.

In Medicare, mean ± SD (median) follow-up was 18.5 ± 11.3 (15.9) months for Black patients and 20.0 ± 11.5 (17.8) months for White patients. Median OS (95% CI) was 44.2 (38.5–not reached [NR]) months for Black patients and NR for White patients. Black patients had a 26% higher unadjusted risk of death vs White patients (Fig. 2; Table 2; HR 1.26, 95% CI 1.16–1.37, *p* < 0.001), and a 20% higher risk of death after adjusting for baseline patient and cancer characteristics (Table 2; HR 1.20, 95% CI 1.09–1.31, *p* < 0.001). In models adding Medicaid enrollment, Black patients had a 13% greater risk of death vs White patients among patients without Medicaid enrollment after adjusting for baseline characteristics (Table 2; Supplementary Fig. S3; median OS 45.5 months vs NR in Black vs White patients, adjusted HR 1.13, 95% CI 1.01–1.27, *p* = 0.037). Among Medicaid-enrolled patients, there was no significant racial difference in OS after adjustments.

However, Medicaid-enrolled patients, regardless of race, had worse OS than patients of the same race who were not Medicaid-enrolled (Table 3; HR 1.50, 95% CI 1.37–1.63, *p* < 0.001 for all patients; HR 1.49, 95% CI 1.31–1.69, *p* < 0.001, and HR 1.57, 95% CI 1.42–1.74, *p* < 0.001 for Black and White patients, respectively).

For the VHA, mean ± SD (median) follow-up was similar for both races: 24.9 ± 15.3 (Black: 21.2; White: 21.4) months. Median OS (95% CI) was 43.6 (38.1–50.3) months for Black patients and 42.2 (39.7–45.5) months for White patients. There was no statistically significant difference in OS between Black and White patients, with or without adjusting for patient characteristics (Fig. 2; Table 2; Supplementary Table S2).

## Discussion

The treatment landscape in mCSPC has changed markedly in recent years [1–9]. Our study is the first to specifically assess emerging racial disparities in TI and OS in mCSPC in the USA, using two large national databases. Given the databases and study size, these results are likely generalizable to patients with mCSPC receiving active treatment in the USA. Moreover, several TI options were available on formulary at the first dates of data collection.

We observed an initial underutilization of TI among the overall population of mCSPC patients, which was more pronounced among Black vs White patients in both Medicare and the VHA. Underutilization of upfront TI is consistent with other reports [14–21]. Our study suggested that underutilization of TI persisted for up to 4 years following the introduction of docetaxel in 2015 and up to 2 years after the introduction of abiraterone in 2017 as life-prolonging therapies for mCSPC. While individual assessment of risk tolerance and goals of care should ultimately determine whether TI is appropriate, these data may signal that urgent action is needed to make mCSPC treatment more guideline-adherent.

Adding to the urgency, Black patients were less likely to receive TI vs White patients after adjusting for differences in baseline characteristics. Less TI for Black vs White patients in Medicare was most evident in patients without Medicaid enrollment (30% less likely for Black patients; Table 2). The racial difference in Medicare patients with Medicaid was not significant (Table 2). For both races, the use of TI was lower among Medicaid-enrolled vs patients who were not Medicaid-enrolled (20% and 37% lower in Black and in White patients, respectively; Table 3), suggesting that economic status affects the use of TI in conjunction with race. The VHA data used for the primary analysis did not contain a variable directly reflecting individual patients’ economic status. However, adjusting for median regional household income and education based on zip code had minimal impact on the racial disparity in TI. This suggests that the economic factors influencing treatment may be mitigated in the VHA, perhaps because of its more uniform benefits design.

In addition, worse OS was observed in Black patients compared with White patients in Medicare overall, as well as among patients without Medicaid enrollment. This disparity in OS was not observed for low economic status or within the VHA, suggesting a potential benefit of a single-payer system with limited out-of-pocket expenses. In the Medicare population, since a greater proportion of Black patients were Medicaid-enrolled vs White patients (40.3% vs 9.0%), the lower OS for Black patients may be at least partially driven by associations with the care of Medicaid-enrolled patients. Indeed, Medicaid-enrolled patients had worse OS compared with patients not enrolled in Medicaid in both races (HR 1.49 for Black patients and 1.57 for White patients, both *p* < 0.001; Table 3), suggesting that poverty, in addition to Black race, is associated with worse OS.

Racial disparities in PC treatment have been demonstrated in prior studies among patients with localized PC or mCRPC [23–30]; however, none have examined disparity among patients with mCSPC during the current NHT era. Our study indicates that racial disparity exists in mCSPC and the number of patients potentially impacted suggests that this is a more extensive problem in mCSPC. Such disparity in Medicare might be partially caused by differences in drug access across races in prescription benefit designs. However, even in a system with a more standardized prescription plan (an equal-access healthcare system, e.g., the VHA), TI disparity by race still exists. The racial disparity of TI in the VHA (25% less likely for Black patients) was in between that observed among Medicare patients with and without Medicaid enrollment. This might be related to physicians’ prescribing habits, and patients’ and physicians’ ability to navigate barriers to obtaining TI, or in some cases patient reluctance.

Previous studies have demonstrated that racial disparities exist in PC outcomes, with Black men experiencing higher mortality rates compared with White men [52]. However, growing evidence suggests that given equal treatment, outcomes may be better for Black than White men with late-stage PC [20, 53–58]. There are limited, inconclusive data on potential differences by race in response to guideline-recommended mCSPC treatment [59–61]. Our findings suggest caution in ruling out racial disparity in either treatment or outcome. While we found that race did not have a significant impact on OS in mCSPC under a system with relatively equal access to care (the VHA) and among the economically disadvantaged (Medicare with Medicaid enrollment), it is not entirely clear if this represents an extension of the findings from the studies examining early disease [40, 42, 59] to mCSPC, or more troublingly, a dampening down of the OS advantage for Black patients in mCRPC studies [53–55, 57], where treatment disparities between races were not as apparent. Moreover, worse OS for Black vs White patients is still observed in the larger subgroup (Medicare without Medicaid enrollment).

Given that we are still early in the era of upfront NHT for mCSPC, future research should elucidate whether racial differences exist in response to TI and, ultimately, patient outcomes. Notably, median OS in our study was around 42 months, coinciding with the ADT-alone arm in CHAARTED [1], LATITUDE [3], TITAN [8], and ARASENS [9] (OS, 36.5–52.2 months). This may be because most patients in both Medicare and the VHA were receiving ADT alone or with NSAA in the current study. If emerging disparities in TI in mCSPC are not addressed aggressively, selective increased use of TI in White patients, including treatment combinations, may further increase disparities in outcomes.

## Limitations

This study is subject to limitations common in retrospective data analyses. First, this study only included patients who received systemic mCSPC treatment; there may be selection bias and findings may not be representative of all patients. Second, given the recent introduction of NHT, TI for mCSPC was low in 2018–2019 in both Medicare and the VHA. Thus, the impact of TI on OS suggested by multiple clinical trials has ostensibly not had a chance to manifest itself, nor is it possible to causally associate the impact of TI, or the lack thereof, with OS outcomes in mCSPC. Third, the Medicare and VHA data used in this study did not include information on some key prognostic factors, rendering it difficult to account for disease risk and volume. Additionally, while we were able to adjust for certain socioeconomic factors, other variables shown to be important to PC survival, such as marital status [62], were not available. Finally, we acknowledge that eligibility criteria for Medicaid differ by state and may have changed over time. Nonetheless, within a given state, those who are Medicaid-enrolled defines a group of patients at the lower end of the socioeconomic spectrum. Further studies using more granular socioeconomic measures as well as social determinants of health are needed to understand which aspects of Medicaid eligibility are driving the associations seen.

## Conclusion

Our study suggests that TI in mCSPC is slowly increasing, but remains low overall, despite unanimous guideline recommendations and evidence of OS improvement. TI is consistently lower in Black vs White patients in both Medicare and the VHA. Race was associated with OS in Medicare but not in the VHA. Importantly, our study shows that Medicare patients who were Medicaid-enrolled received less TI and had worse OS in mCSPC. This suggests that poverty, in addition to race, is associated with quality of care and outcomes. It is concerning that treatment disparities and potentially worse survival outcomes are emerging in mCSPC when life-prolonging treatments are available and established as the standard of care. There is an important role for guideline committees and healthcare practitioners, as well as population-based decision-makers such as those overseeing treatment pathways and algorithms, in ensuring that TI is provided, as appropriate, to patients with mCSPC, regardless of economic status or race.

## Supplementary information

Supplementary Material

Video Abstract

## Data Availability

As the data supporting the findings of this study were used under license for the current study, restrictions apply to the authors’ ability to make data publicly available. The data are available from the Research Data Assistance Center and the Veterans Health Administration.
