# Supplementary material for: Emerging racial disparities among Medicare beneficiaries and Veterans with metastatic castration-sensitive prostate cancer
Source: Prostate Cancer Prostatic Dis. 2024 Apr 2;27(4):765–75. doi: 10.1038/s41391-024-00815-1 (PMC11543599; doi:10.1038/s41391-024-00815-1)
Supplement: Supplementary file 1 — Supplementary Material [file 41391_2024_815_MOESM1_ESM.docx]

# Supplementary information

Table S1. Additional patient baseline characteristics in the Veterans Health Administration dataset.

|  | **Overall (*N* = 3 384)** | **Black patients (*n* = 1 020)** | **White patients (*n* = 2 364)** | **Black vs White patients SMD, %** |
| --- | --- | --- | --- | --- |
| PSA, *n* (%) | 3 384 (100) | 1 020 (100) | 2 364 (100) | 0.00 |
| PSA, mean ± SD (median) [IQR] | 222.9 ± 1045.6  (27.7) [6.1–137.0] | 345.4 ± 1714.6  (40.5)  [7.7–213.3] | 170.1 ± 536.7  (23.6)  [5.6–113.6] | 13.80 |
| Hemoglobin, *n* (%) | 2 950 (87.2) | 910 (89.2) | 2 040 (86.3) | 8.92 |
| Hemoglobin, mean ± SD (median) [IQR] | 12.8 ± 2.2  (13.0)  [11.4–14.4] | 12.1 ± 2.2  (12.4)  [10.6–13.6] | 13.1 ± 2.1  (13.3)  [11.8–14.6] | –47.06 |
| Alkaline phosphatase, *n* (%) | 2612 (77.2) | 792 (77.6) | 1820 (77.0) | 1.57 |
| Alkaline phosphatase, mean ± SD (median) [IQR] | 200.4 ± 335.4  (94.0)  [71.0–161.5] | 201.1 ± 280.5  (94.0)  [72.0–187.0] | 200.1 ± 356.8  (94.0)  [70.0–156.0] | 0.33 |
| Household income^a^, mean ± SD (median) [IQR] | 61 893 ± 23 523  (57 132)  [45 969–73 075) | 54 230 ± 21 392  (50 260)  [39 412–64 626] | 65 173 ± 23 635  (60 134)  [49 347–76 294] | –48.55 |
| Percent of population age ≥25 with bachelor’s degree per zip code^a^, mean ± SD (median) [IQR] | 28.5 ± 14.8  (25.0)  [17.5–36.1] | 26.6 ± 14.3  (23.5)  [16.1–33.6] | 29.3 ± 14.9  (25.7)  [18.3–37.6] | –18.99 |

^a^Obtained from American Community Survey 2019. The household income was imputed using the 5-year estimates for median household income per zip code. *IQR* interquartile range, *PSA* prostate-specific antigen, *SD* standard deviation, *SMD* standardized mean difference.

Table S2. Rate of first-line treatment intensification and OS for mCSPC^a^ among Black and White patients in Veterans Health Administration data, adjusting for additional variables.

|  | **Black vs White patients** | ***P*-value** |
| --- | --- | --- |
| Adjusted model 2 (Table 2) + additionally adjusting for log(PSA), hemoglobin, and log(alkaline phosphatase) | | |
| Intensification of first-line treatment for mCSPC, OR (95% CI) | 0.65 (0.51–0.84) | < 0.001* |
| OS, HR (95% CI) | 0.95 (0.82–1.10) | 0.482 |
| Adjusted model 2 (Table 2) + additionally adjusting for median income and educational attainment^b^ | | |
| Intensification of first-line treatment for mCSPC, OR (95% CI) | 0.77 (0.63–0.95) | 0.017* |
| OS, HR (95% CI) | 1.07 (0.94–1.22) | 0.296 |

^a^Intensification of first-line treatment for mCSPC was defined as treatment with androgen deprivation therapy + novel hormonal therapy or androgen deprivation therapy + docetaxel.
^b^For each patient, the median household income in the zip code was used for adjustment. Education attainment was the percentage of population in a zip code age 25+ with at least a bachelor’s degree or higher.
*CI* confidence interval, *HR* hazard ratio, *mCSPC* metastatic castration-sensitive prostate cancer, *OR* odds ratio, *OS* overall survival, *PSA* prostate-specific antigen.

Table S3. Distribution of the subsequent treatment.

| **n (%)** | **Medicare** | | **VHA** | | |
| --- | --- | --- | --- | --- | --- |
|  | **Black patients (*n* = 2 226)** | **White patients (*n* = 16 071)** | **Black patients (*n* = 1 020)** | **White patients (*n* = 2 364)** |  |
| No subsequent treatment^a^ | 1 590 (71.4) | 11 300 (70.3) | 609 (59.7) | 1364 (57.7) |  |
| NHT | 310 (13.9) | 2 273 (14.1) | 261 (25.6) | 650 (27.5) |  |
| NSAA | 201 (9.0) | 1269 (7.9) | 81 (7.9) | 209 (8.8) |  |
| Chemotherapy | 96 (4.3) | 797 (5.0) | 37 (3.6) | 70 (3.0) |  |
| NHT + chemotherapy | 0 (0) | 0 (0) | 20 (2.0) | 41 (1.7) |  |
| Other^b^ | 29 (1.3) | 432 (2.7) | 12 (1.2) | 30 (1.3) |  |

^a^Includes patients still on first-line treatment.
^b^“Other” included sipuleucel-T, radium-223, olaparib, and combination therapies used by less than 11 patients.
*NHT* novel hormonal therapy, *NSAA* nonsteroidal antiandrogen, *VHA* Veterans Health Administration.

Fig. S1. Sample selection.


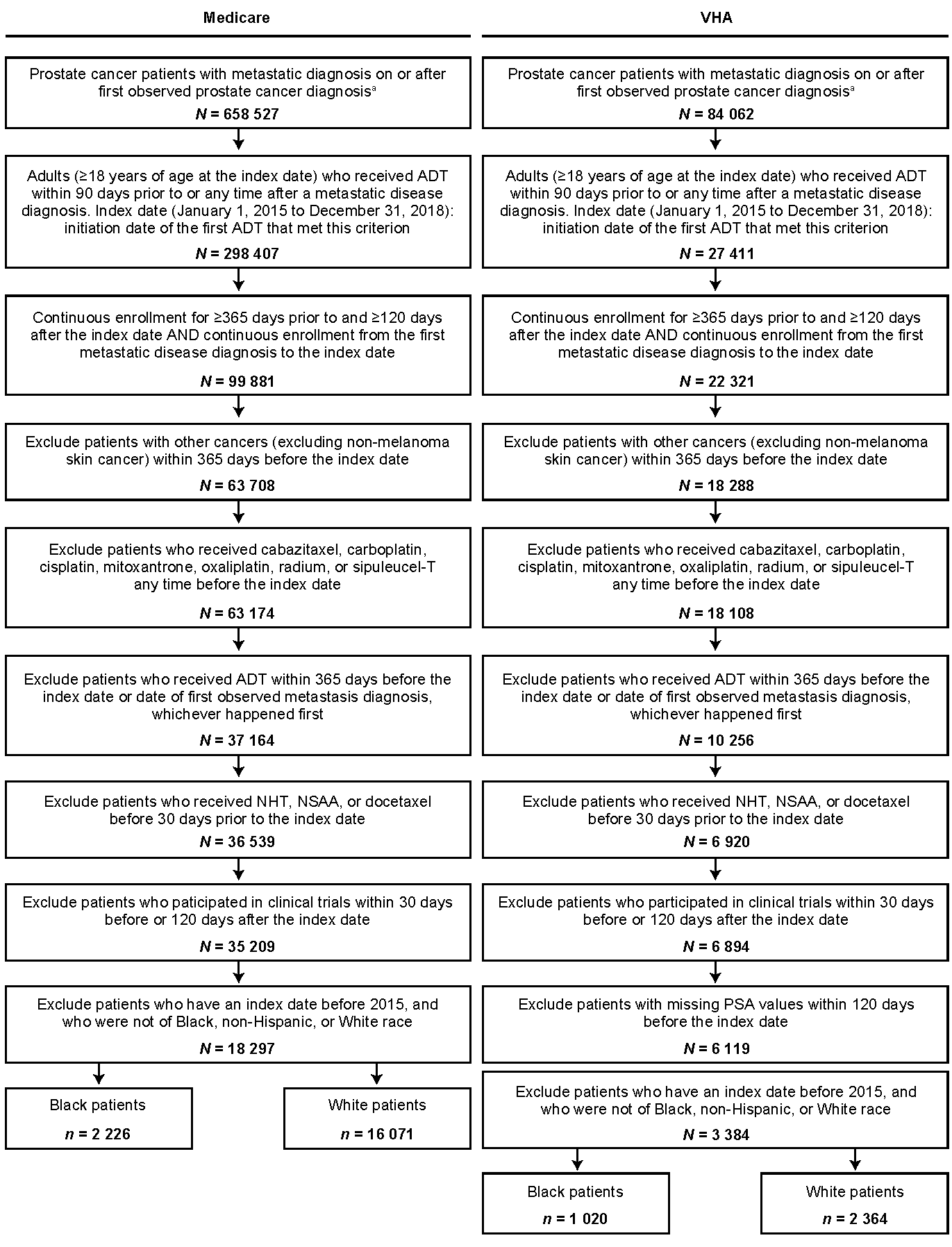


^a^Patients were included in the study if they had at least one medical claim with an International Classification of Disease, Ninth/Tenth Revision, Clinical Modification (ICD-9/10-CM) diagnosis code for prostate cancer (ICD-9-CM: 185; ICD-10-CM: C61), and had at least one medical claim for metastasis (ICD-9-CM: 196-199.1; ICD-10-CM: C77, C78, C79, C7B) on or after the first observed prostate cancer diagnosis.

*ADT* androgen deprivation therapy, *CM* clinical modification, *ICD* International Classification of Disease, *NHT* novel hormonal therapy, *NSAA* nonsteroidal antiandrogen, *PSA* prostate-specific antigen, *VHA* Veterans Health Administration.

Fig. S2. First-line treatment for mCSPC over time by race among Medicare patients (A) with Medicaid enrollment (B) without Medicaid enrollment.


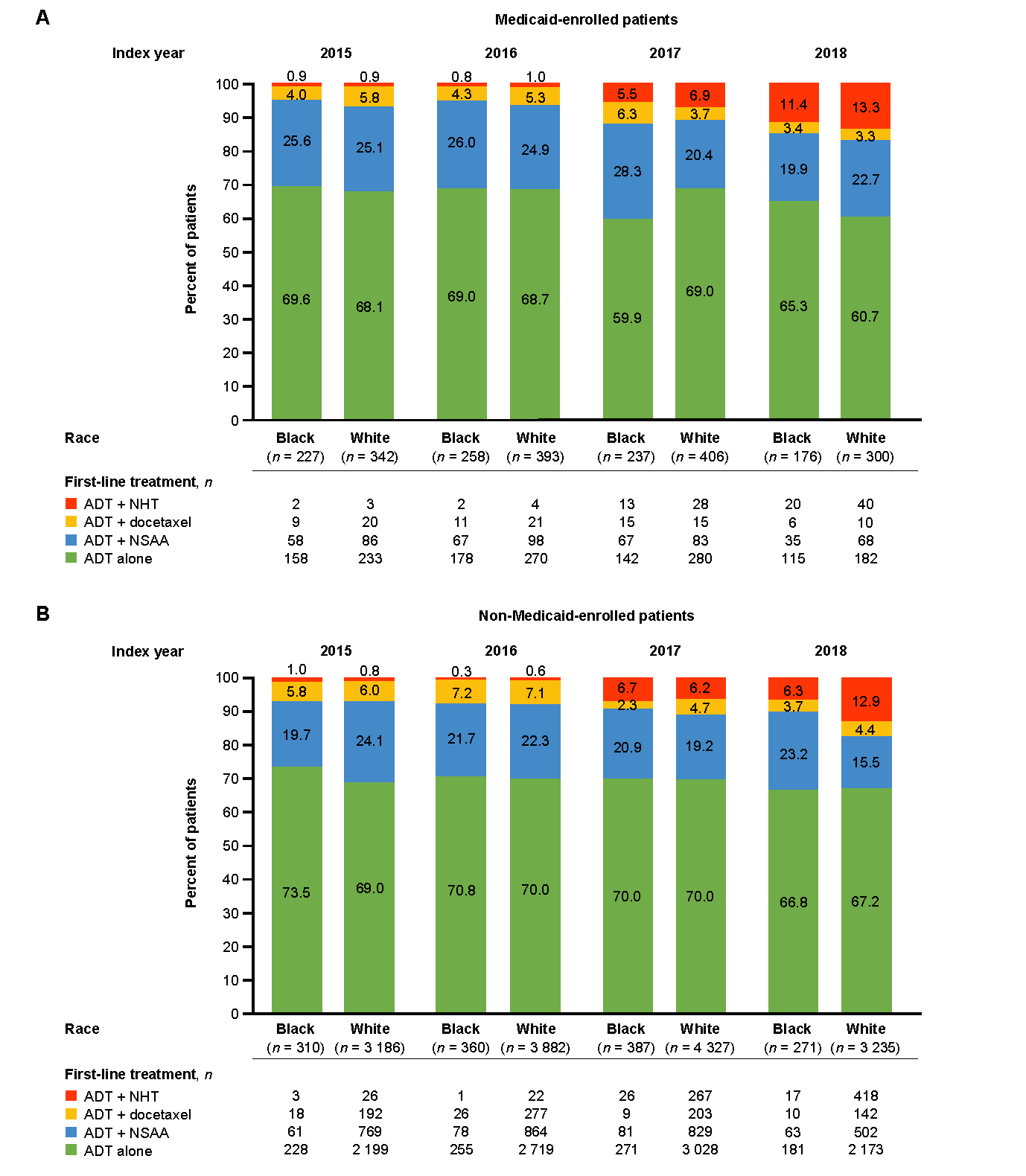


*ADT* androgen deprivation therapy, *mCSPC* metastatic castration-sensitive prostate cancer, *NHT* novel hormonal therapy, *NSAA* nonsteroidal antiandrogen.

Fig. S3. OS by race among Medicare patients with mCSPC (A) with Medicaid enrollment (B) without Medicaid enrollment.

**
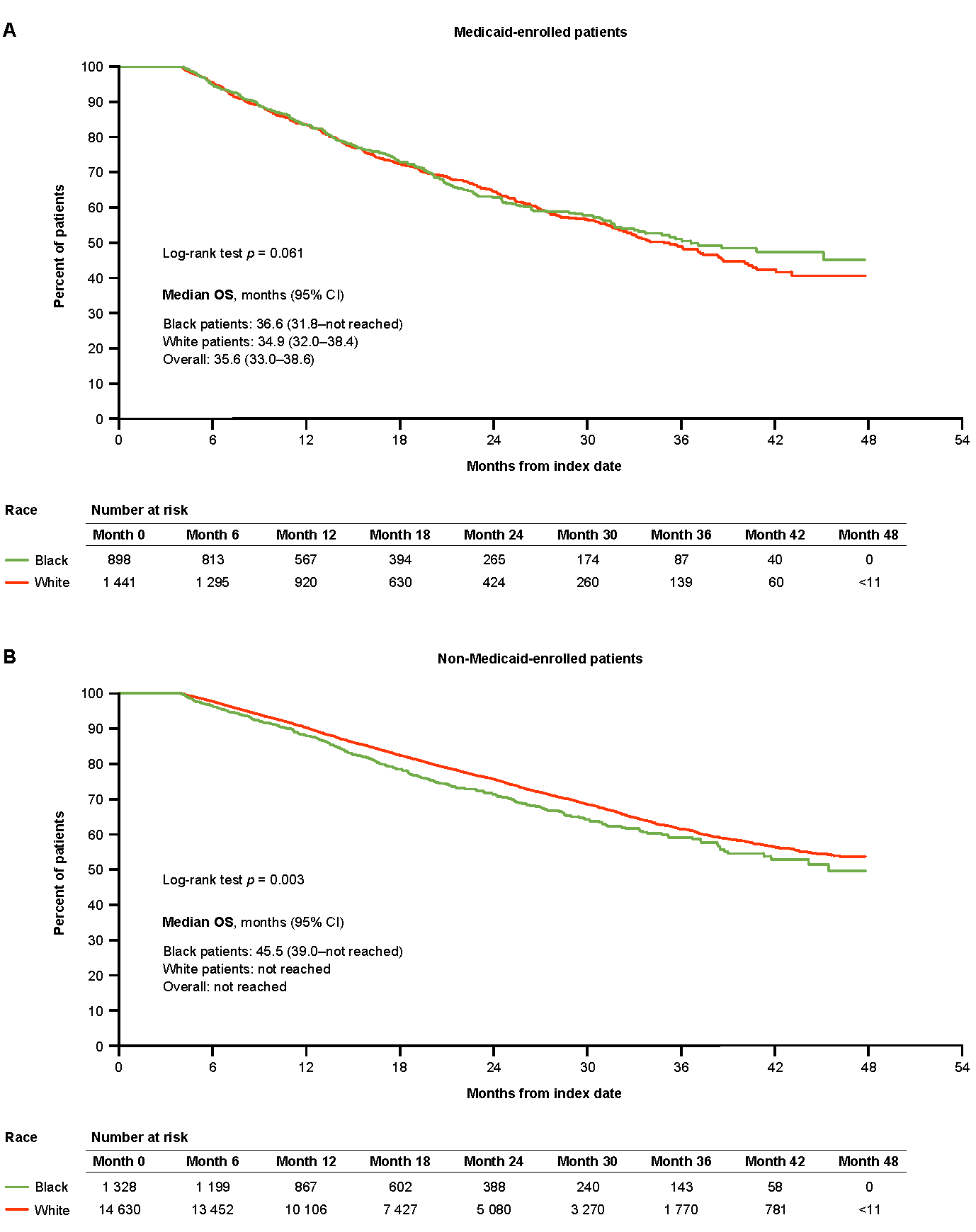
**

*CI* confidence interval, *mCSPC* metastatic castration-sensitive prostate cancer, *OS* overall survival.
